# Rare Chance

**Published:** 1878-11-20

**Authors:** 


### RARE CHANCE!

Notice advertisement headed *A Rare Chance*, as it furnishes a splendid opening for some man.

